# Lateral Tunnel Epitaxy of GaAs in Lithographically Defined Cavities on 220 nm Silicon-on-Insulator

**DOI:** 10.1021/acs.cgd.3c00633

**Published:** 2023-10-12

**Authors:** Zhao Yan, Bogdan-Petrin Ratiu, Weiwei Zhang, Oumaima Abouzaid, Martin Ebert, Graham T. Reed, David J. Thomson, Qiang Li

**Affiliations:** # School of Physics and Astronomy, Cardiff University, Cardiff CF24 3AA, U.K.; ‡ Optoelectronics Research Centre, University of Southampton, Southampton SO17 1BJ, U.K.

## Abstract

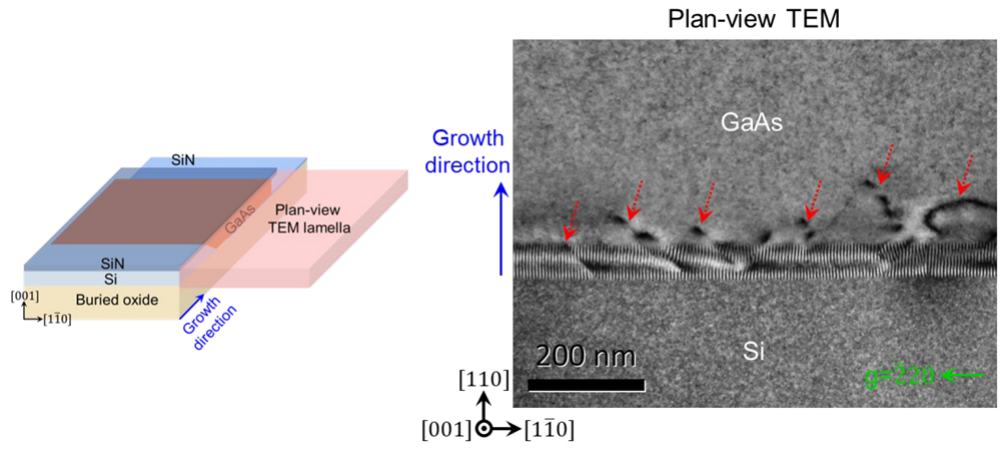

Current heterogeneous Si photonics usually bond III–V wafers/dies on a silicon-on-insulator (SOI) substrate in a back-end process, whereas monolithic integration by direct epitaxy could benefit from a front-end process where III–V materials are grown prior to the fabrication of passive optical circuits. Here we demonstrate a front-end-of-line (FEOL) processing and epitaxy approach on Si photonics 220 nm (001) SOI wafers to enable positioning dislocation-free GaAs layers in lithographically defined cavities right on top of the buried oxide layer. Thanks to the defect confinement in lateral growth, threading dislocations generated from the III–V/Si interface are effectively trapped within ∼250 nm of the Si surface. This demonstrates the potential of in-plane co-integration of III–Vs with Si on mainstream 220 nm SOI platform without relying on thick, defective buffer layers. The challenges associated with planar defects and coalescence into larger membranes for the integration of on-chip optical devices are also discussed.

## Abstract 2

Lateral tunnel epitaxy enables dislocation-free III−V membranes in close proximity to silicon for on-chip III−V/Si photonic integration on the 220 nm silicon-on-insulator platform.

## Introduction

Developing photonic integrated circuits (PICs) is not only essential to meet the ever-growing demand for high speed, low power short-reach data communications, but also instrumental in emerging consumer-orientated optical applications requiring high scalability, functionality and reliability.^1−3^ While Si photonics platforms provide low-cost, high volume manufacturability by leveraging CMOS-compatible foundry processes, heterogeneous integration of direct bandgap III–V materials onto the Si photonic chips can address the deficiencies of silicon in making light sources and significantly increase the data transmission capacity of optical modulators.^2−4^ To date, high performance devices have been demonstrated by bonded III–V wafers/dies on silicon-on-insulator (SOI) substrates.^5^ A truly monolithic photonic integration platform is highly desirable for scalable, cost-effective and densely integrated Si-based PICs.^2,3^ Epitaxial growth of GaAs and InP, the two most important active materials for visible and near-infrared communication wavelengths, onto the silicon platform has been extensively pursued. Conventional buffer layer epitaxy, which often involves a few-micron thick transitional buffers on bulk silicon or patterned SOI substrates, still yields threading dislocation (TD) densities on the order of 10^6^ cm^–2^ or above.^6,7^ This has to be combined with dislocation tolerant materials such as quantum dots in order to meet the stringent lifetime requirement for telecom wavelength lasers.^8,9^ Efficient coupling from the epitaxial lasers to Si waveguides remains a critical challenge to date.^10,11^

Recent development in lateral epitaxy, a growth technique also demonstrated in Si tunnel epitaxy,^12^ has shown the potential of growing “buffer-less” dislocation-free III–V materials on SOI substrates.^13−18^ It brings the III–V optical active region in close proximity to Si which has enabled high-performance waveguide coupled photodetectors.^19,20^ For the integration of light sources on silicon, the most progress so far has been made on optically pumped nanolasers with small device footprints and limited active materials.^21,22^ It remains a significant challenge to achieve large-area dislocation-free III–V materials suitable for electrically injected edge emitting laser devices to produce sufficient output power. Here, we demonstrate a front-end-of-line (FEOL) patterning and epitaxy process on Si photonic 220 nm SOI substrates and present a materials study of GaAs growth in lithographically defined cavities. The growth cavity was designed to have a length of 150 μm predefined by lithography for potential edge emitting laser applications. Lateral tunnel epitaxy of GaAs was performed by using selective-area metal–organic chemical vapor deposition (MOCVD). The defect necking mechanism is verified by plan-view and cross-sectional transmission electron microscopy (TEM) investigations. We found that TDs originated from the Si surface can be effectively trapped within ∼250 nm lateral distance. Despite twinning segments in the coalescence, defect-free GaAs regions up to a few microns in length were revealed in TEM. These results show the potential of developing such a material platform for high modulation speed membrane lasers with tight optical confinement inside the III–V layer and facilitate efficient light interfacing to Si-based passive devices.^23^

## Results

**Figure 1:**
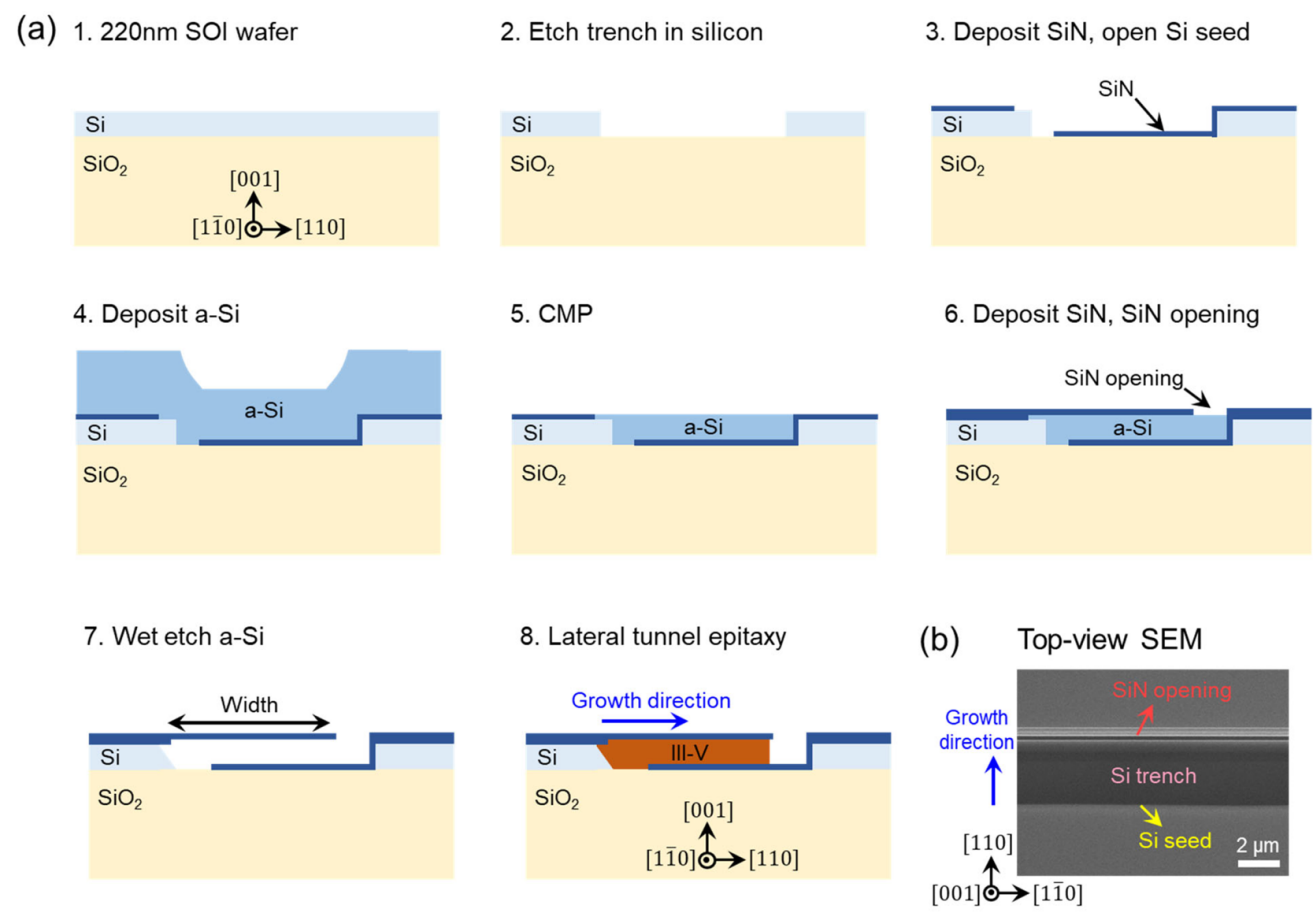
(a) Process flow of the front-end process for lateral tunnel epitaxy on a 220 nm SOI. (b) Top-view SEM photo of fabricated SOI patterns. The top SiN layer is transparent under top-view SEM.

As shown by the schematic in Figure 1a, we first developed a front-end patterning and epitaxy process on a standard 220 nm SOI substrate that allows the selective growth of GaAs prior to the formation of Si waveguides or metallization. Insertion of III-Vs on SOI at an early stage can bring out two advantages: First, selective growth of III–V materials in lithographically defined cavities can remove the challenges in aligning III–V and Si waveguides in the bonding approach, and co-fabrication of the III–V/Si devices can be performed to realize integrated optical functionalities; Second, the high temperature III–V growth is performed in advance of other device formation and metallization steps which is beneficial for the thermal budget of Si devices. Starting from an 8-inch 220 nm (001) SOI substrate, etching Si trenches was carried out in step 2 (Figure 1a). A 40 nm SiN layer was deposited and patterned to open a Si seed at one side of the trench (step 3). A thick amorphous Si (a-Si) layer was then deposited and planarized by chemical mechanical polishing (CMP). Note that the predeposited 40 nm SiN can be used here as an etch stop layer for the CMP process in step 5. Afterward, another 200 nm SiN layer was deposited, and the growth opening in the SiN layer were defined at the other side of the Si trench (step 6). The a-Si sacrificial layer was then removed by TMAH wet etch. This was then followed by a hydrofluoric acid (HF) cleaning step to remove any native oxide present between the a-Si and the crystalline Si layer. A further TMAH etch was performed to produce smooth {111}-orientated Si facets at the Si seed (step 7), which is important to prevent antiphase boundaries (APBs) in III–V/Si heteroepitaxy.^24,25^ Finally, the lateral tunnel epitaxy of III-Vs was carried out in MOCVD (step 8). To employ the III–V layer for practical device applications, the top SiN layer can be removed by either HF wet etching or dry etching methods, such as inductively coupled plasma (ICP). Figure 1b displays the top-view SEM photo of the fabricated SOI pattern: the top SiN layer is transparent, and the Si trench and Si seed underneath are visible. As indicated by the blue arrow of growth direction, III–V lateral tunnel epitaxy will initiate from the Si seed and grow in the lateral direction parallel to substrate surface.

**Figure 2:**
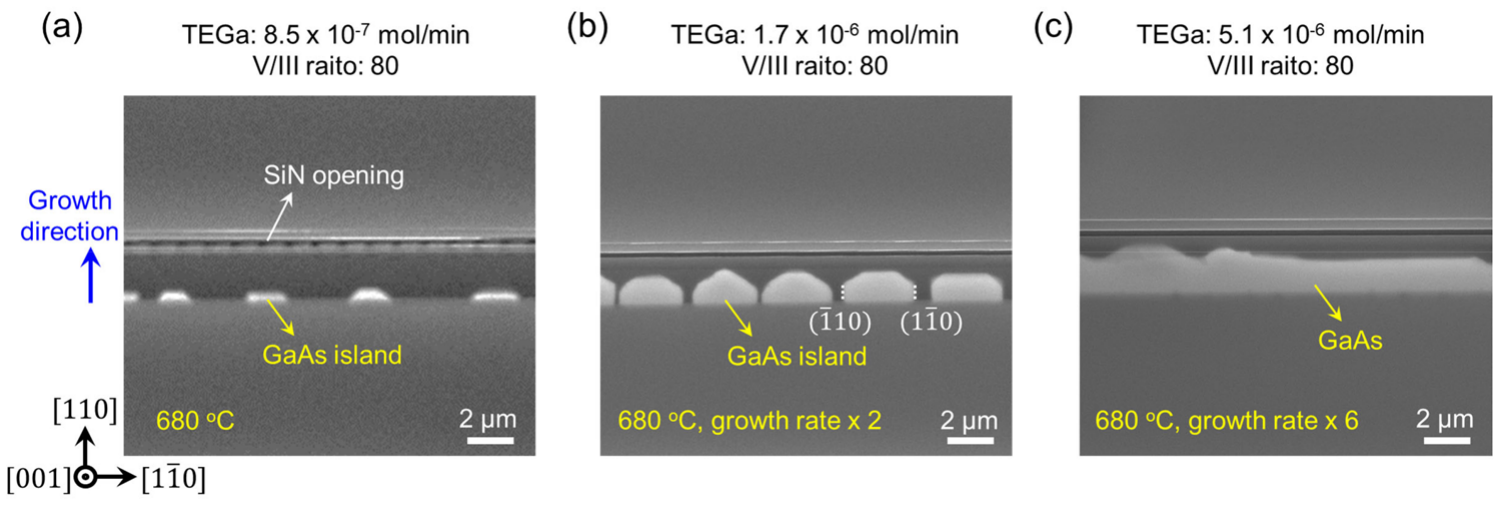
Top-view SEM photos of as-grown GaAs inside the tunnel without the low temperature GaAs nucleation layer. The top SiN layer is transparent under top-view SEM, so the GaAs underneath is visible. (a) Tiny GaAs islands deposited at a slow growth rate. (b) Larger GaAs islands with clear faceting are formed. The GaAs islands exhibit two {110} facets on both sides. (c) GaAs grown inside the tunnel at increased growth rate. The GaAs growth front formed both straight and bumpy end facets.

Lateral tunnel epitaxy of GaAs was performed using MOCVD with H_2_ as the carrier gas and triethylgallium (TEGa) and tertiarybutylarsine (TBAs) as growth precursors. Prior to growth, the sample was dipped in diluted HF to remove the native oxide on the Si seed surface and then immediately loaded into the MOCVD reactor. The growth process started with 800 °C annealing for 20 min to thermally desorb any residual oxide on the {111} Si facets. GaAs was then grown at a single temperature of 680 °C. In conventional blanket heteroepitaxy of GaAs thin films on planar Si substrates, a nucleation layer deposited at a low temperature (LT) was commonly used prior to the main layer grown at typical high temperatures.^25^ In this study, such a LT nucleation layer was not used in order to achieve an adequate growth selectivity (as shown by the large-scale microscope images of Figure S1a for detailed discussions). As displayed by the top-view SEM photo in Figure 2a, by applying a low precursor flow, GaAs islands were formed spreading out along the (111) Si surface. By increasing the growth rate, denser GaAs islands emerge with well-developed crystalline facets (see Figure 2b). The two vertical {110} facets are formed at both sides of the GaAs islands as indicated by the white dotted line in Figure 2b, similar to other selective growth of III–V nanoridges and vertical III–V nanowires with six {110} side-facets.^26,27^ By further increasing of the growth rate, a continuous GaAs layer could be obtained, as indicated by Figure 2c. The GaAs growth time was kept at 60 min in these three samples.

**Figure 3:**
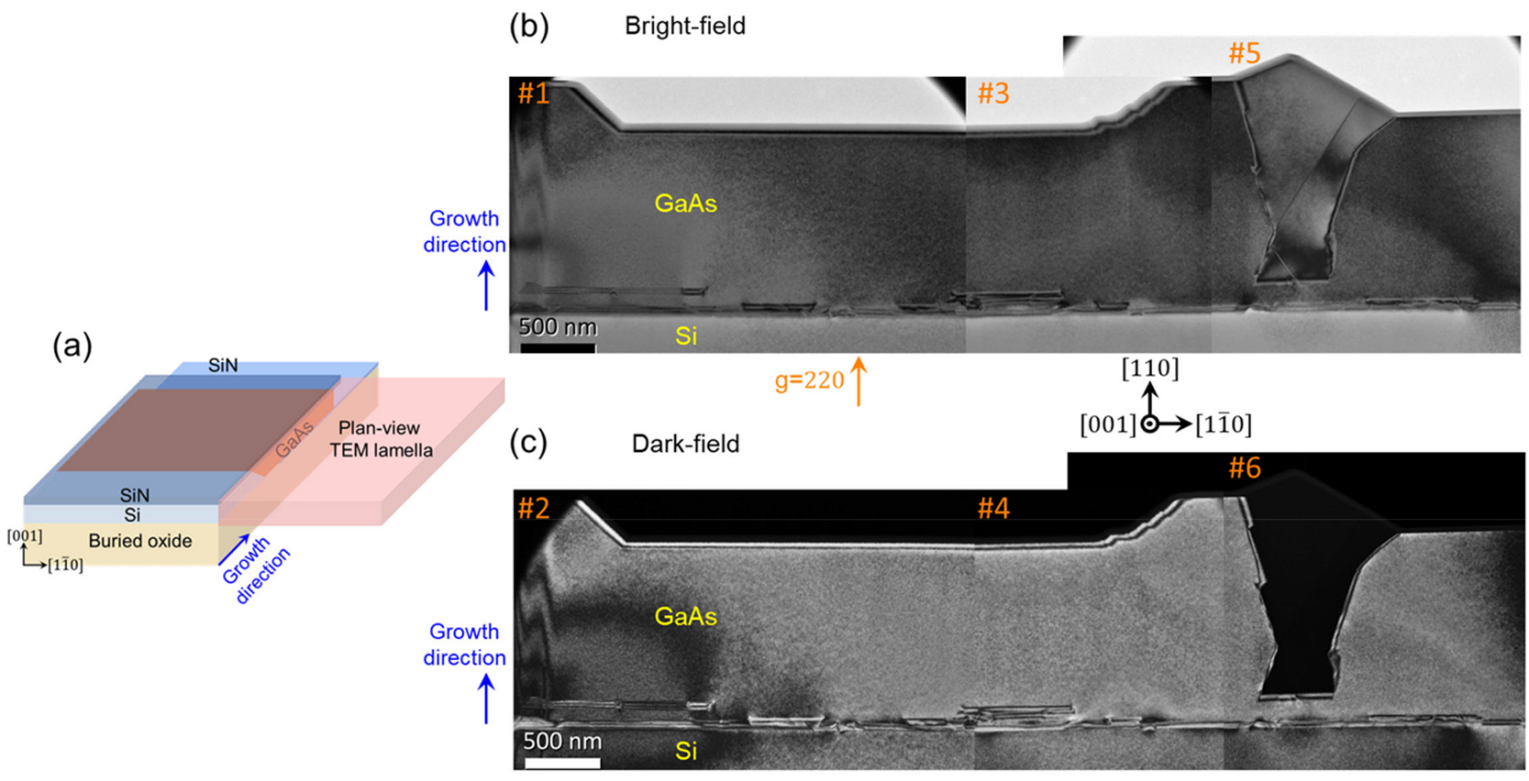
Plan-view TEM montage of the one-step GaAs laterally grown on SOI without a low-temperature GaAs nucleation layer (sample as shown in Figure 2c). (a) A schematic illustrates the orientation of the plan-view TEM lamella along the [001] zone axis. The TEM lamella incorporates the entire GaAs layer. (b) Bright-field and (c) dark-field TEM montages including both the Si and GaAs layer. The original TEM images 1–6 were acquired under a *g* vector = 220 diffraction condition.

We prepared plan-view GaAs/Si TEM lamella by using focused ion beam (FIB) milling. The schematic of Figure 3a illustrates the orientation of the TEM lamella which includes both the Si layer and the in-plane GaAs layer. The thickness of the TEM lamella was approximately 400 nm. During the ion milling process, intended to thin the TEM lamella, the SiN layer was still present as well as SiO_2_ on the opposite face. As such, the TEM lamella incorporates SiN and SiO_2_ on both sides and the epitaxial GaAs in the middle. This is quite thick for TEM, but still yields acceptable images aided by the anomalous transmission effect when a two-beam diffraction condition is used. The TEM montages in Figures 3b and 3c present paired bright-field and dark-field images taken in the *g* = 220 diffraction condition. GaAs regions with both straight and bumpy growth fronts can be observed. The bumpy growth front likely results from the coalescence of two adjacently situated, non-{110}-orientated side-facets of GaAs islands. The dark area near the bumpy growth front in Figure 3c suggests it has a twinned orientation different from the surrounding GaAs, which could be associated with the planar defects in this same area. A cross-sectional-TEM analysis of a similar twinned section will be presented later. Despite this problematic coalescence region, Figure 3 shows clean GaAs membrane region with length of several microns that are completely free of any crystalline defects, which is not feasible in conventional thin film heteroepitaxy.

**Figure 4:**
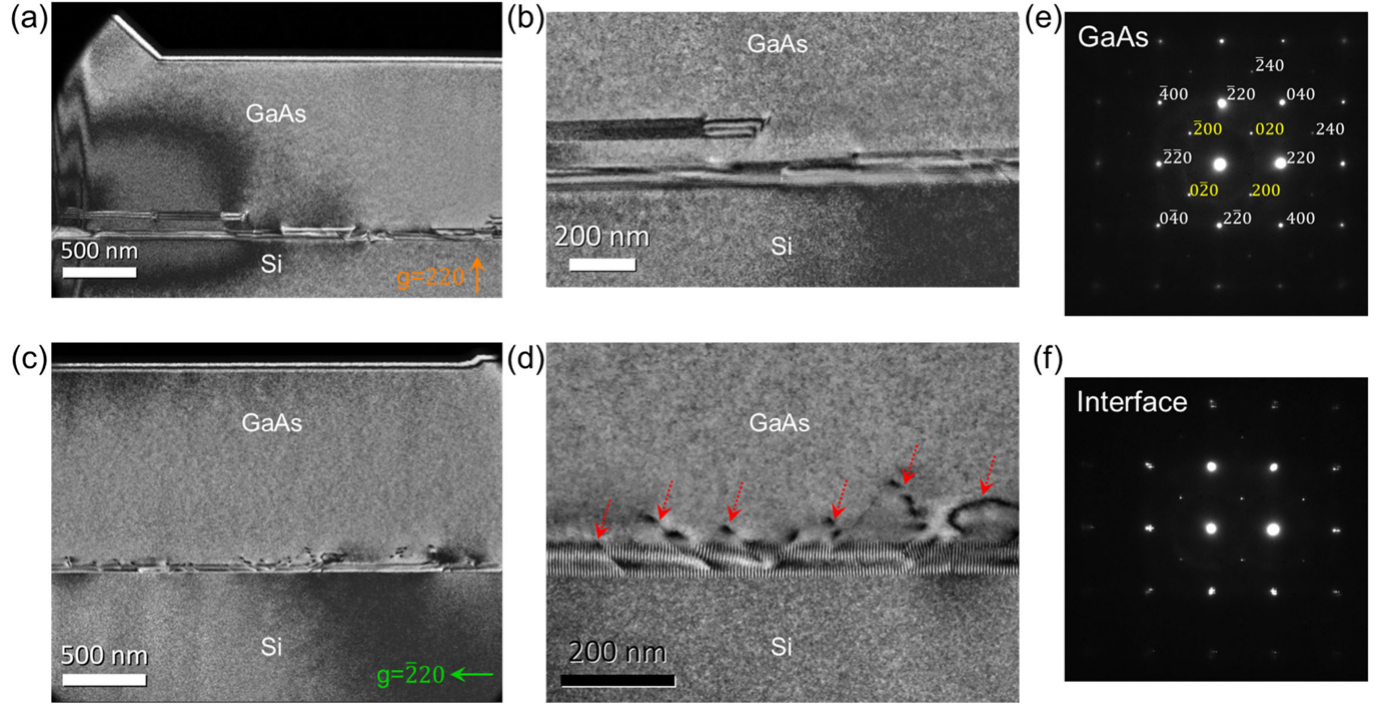
Plan-view TEM analysis for the confinement of dislocations and planar defects in laterally grown GaAs on SOI. (a) Dark-field plan-view TEM image (*g* = 220) encompasses both Si and GaAs region. (b) An enlarged TEM image reveals planar defects confined near the Si region. (c) Dark-field plan-view TEM image (*g* = 220) showing the confinement of dislocations near the Si interface. (d) A zoomed-in TEM image to show the propagation and effective trapping of dislocations. Plan-view electron diffraction patterns of the (e) GaAs and (f) Si/GaAs interface, respectively.

More TEM investigations were conducted near the Si interface to understand the defect trapping effect from the lateral tunnel epitaxy. Using the same *g* = 220 condition, Figure 4a–b shows zoomed-in plan-view TEM images where planar defects close to the Si region were observed. These planar defects are stacking faults (SFs) and twins running parallel to the {111} planes and they terminate at the top SiN layer and buried oxide layer in a short distance from the Si seed. Figure 4c and 4d present the similar region but using the perpendicular *g* = 220 diffraction condition. Dislocations, which were invisible under the previous *g* = 220 diffraction condition in Figures 3 and 4a, now become visible with bright and dark contrast on the dislocation lines. As indicated by the red arrow in Figure 4d, this contrast is due to dynamical diffraction of electron beams in the TEM and indicates the dislocation lines are inclined through the TEM lamella.^28^ In the same region, uniform moiré fringe can be seen due to the overlap between Si and GaAs lattices. The clearly distinguished moiré fringe indicates relaxation of the GaAs layer. In both *g* = 220 and *g* = 220 conditions, all these crystalline defects are well confined within ∼250 nm distance from the Si seed. Beyond this localized defective interface, the GaAs main layer is free of any threading dislocations. The crystalline quality of the epitaxial GaAs was further examined by selective diffraction patterns taken from the GaAs and Si/GaAs interface along the [001] zone axis, as shown in Figure 4e and 4f. The kinematically forbidden 002 reflections which are absent in the Si diffraction pattern appear dim in GaAs due to the two sublattices being occupied by group III and group V elements.^29^ At the Si/GaAs interface, both the Si and GaAs diffraction spots are noticeable, accompanied by some double diffraction, which manifests a little grid at each *g*-vector.

**Figure 5:**
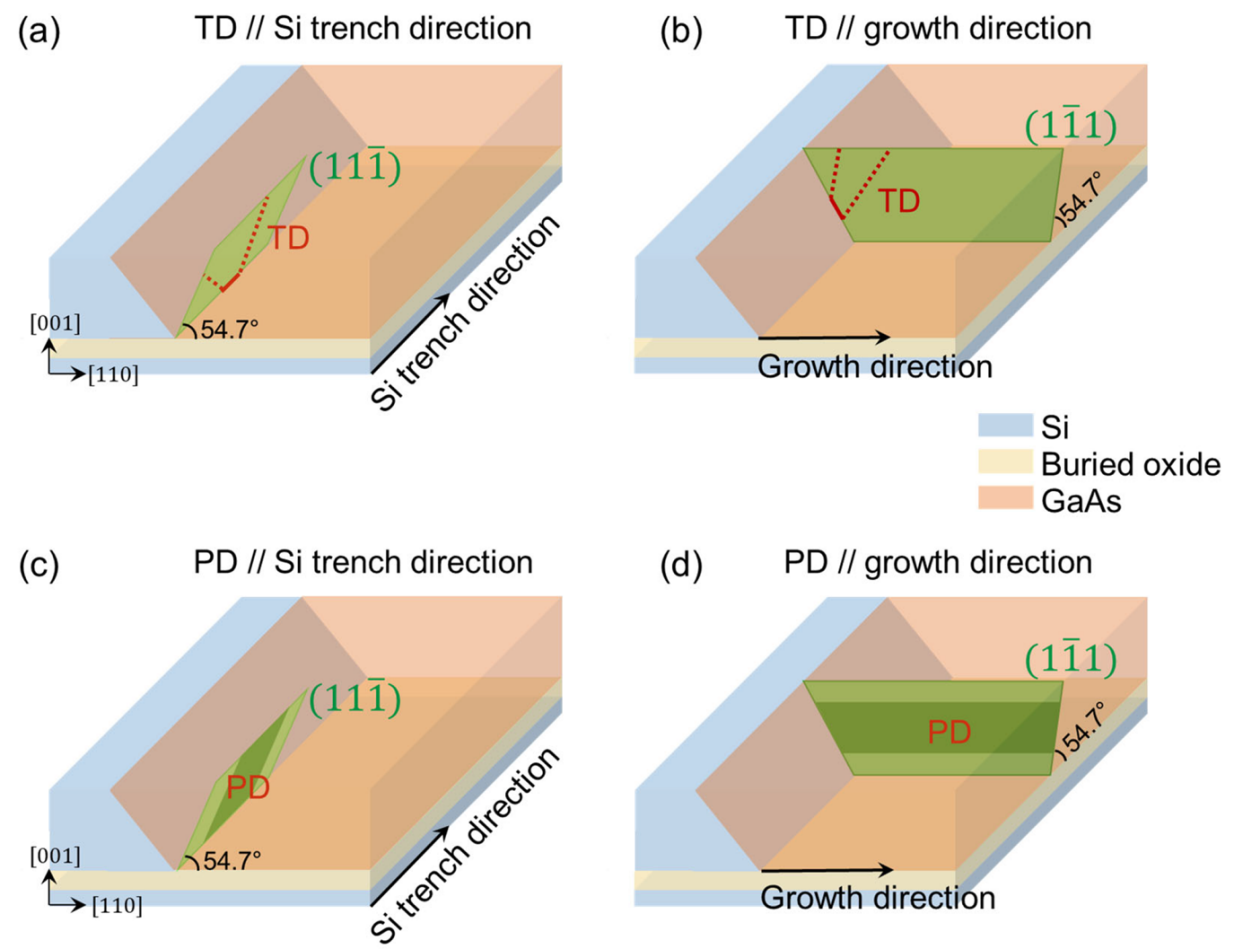
Schematic showing the confinement of dislocations and planar defects in lateral tunnel epitaxy. TDs propagating along the {111} plane parallel to the Si trench direction (a) and along the {111} plane parallel to the growth direction (b) can be trapped. (c) Planar defects at the {111} plane parallel to the Si trench direction can be trapped. (d) Planar defects at the {111} plane parallel to the growth direction cannot be trapped.

The schematics in Figure 5a and 5b summarize the propagation and confinement of dislocations. In GaAs and InP grown on (001) Si, strain can be released through nucleating dislocation half-loops close to Si interface. The dislocation half-loops will then expand in width and glide downward to the Si surface along the {111} slip plane.^26^ In this process, two threading segments are formed (see red dotted line in Figure 5a and 5b) and will continue to propagate upward along the {111} slip plane during the growth process. The dislocations formed are 60° dislocations whose Burgers vector is 60° to dislocation line vector, which are the most common type in GaAs and InP grown on (001) Si.^30^ The {111} slip planes are indicated as the green plane in Figure 5a and 5b. For TDs gliding along the {111} plane parallel to the Si trench direction (see Figure 5a), they will terminate at the top SiN layer, as the {111} plane parallel to the Si trench direction itself intrinsically does not extend into the GaAs main layer. For TDs gliding at the {111} plane parallel to the growth direction (see Figure 5b), the dislocation trapping mechanism also works. While GaAs growing in the lateral direction, the inclined TDs propagate upward (in [001] direction) and will finally hit the top SiN layer. The trapping distance of TDs relies on the crystalline planes and is generally 1.4 times to the thickness of the Si device layer, similar to aspect ratio trapping.^31^ The 220 nm Si device layer here (or height of the growth cavity) translates into a dislocation trapping distance of ∼310 nm, which reasonably agrees with the observed distance of ∼250 nm in our plan-view TEM. The effective confinement of TDs in the in-plane direction highlights the bufferless feature of lateral tunnel epitaxy and its potential for Si-based optoelectronic integrations in close proximity to Si.

The schematics of Figure 5c and 5d summarize the situation of planar defects in lateral tunnel epitaxy. Planar defects such as stacking faults (SFs) and twins run parallel to the {111} planes. For planar defects at the {111} planes parallel to the Si trench direction (Figure 5c), as observed in the TEM image of Figure 4a, 4b, they will terminate at the top SiN layer and buried oxide layer and thus are confined near the Si seed. However, planar defects running parallel to the growth direction (Figure 5d) cannot be trapped. Different from stacking faults in conventional III–V buffers on silicon which are often accompanied by two partial dislocations at both ends, the planar defect in Figure 5d tends to terminate at the free surface of the dielectric layer and thus avoids introducing dangling bonds inside the III–V material.^32^ As a result, their impact on device performance could be less severe. Clean and smooth {111} Si growth surfaces and optimized growth conditions could also help reduce the density of planar defects along this direction.^26^

**Figure 6:**
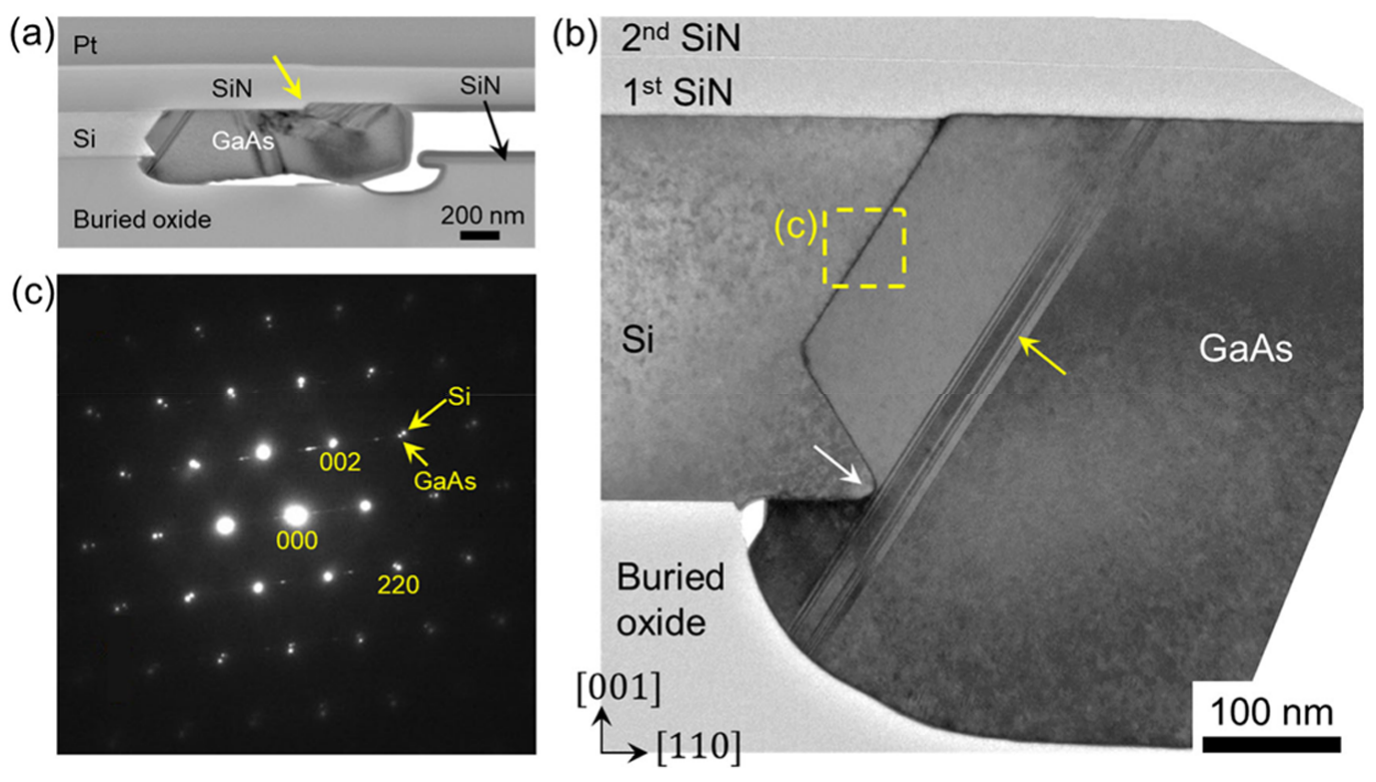
Cross-sectional TEM characterization of GaAs laterally grown on SOI. (a) Global-view and (b) zoomed-in TEM images. (c) Electron diffraction patterns showing both the Si and GaAs lattices, indicating the zincblende phase of GaAs.

Figure 6a shows a cross-sectional TEM image along the lateral growth direction, where planar defects are present. A further zoomed-in image near the GaAs-Si interface is given in Figure 6b. In this particular sample, an unintentional over etch from HF resulted in an uneven SiO_2_ surface beneath the GaAs. The yellow arrow in Figure 6a indicates a step at the bottom surface of the upper SiN layer, a structural imperfection from pattern fabrication. Planar defects appear to form near this area, a phenomenon often observed in other selective area heteroepitaxy.^33,34^ In Figure 6b, the over etch of the buried oxide created a silicon corner (indicated by the white arrow in Figure 6b) and an undercut region. As a result, GaAs was able to grow under the bottom (001) Si plane. Planar defects form at the tip of the Si corner, running parallel to the {111} Si surface and terminating at the top SiN and bottom buried oxide layer. These observations highlight the impact of structural and morphological irregularities of growth patterns on generating new defects during lateral epitaxy, particularly stacking faults and twin defects. Figure 6c shows an electron diffraction image at the GaAs/Si interface with the Si and GaAs diffraction spots marked along with the associated orientation of crystal planes, confirming the zincblende phase of the epitaxial GaAs.

**Figure 7:**
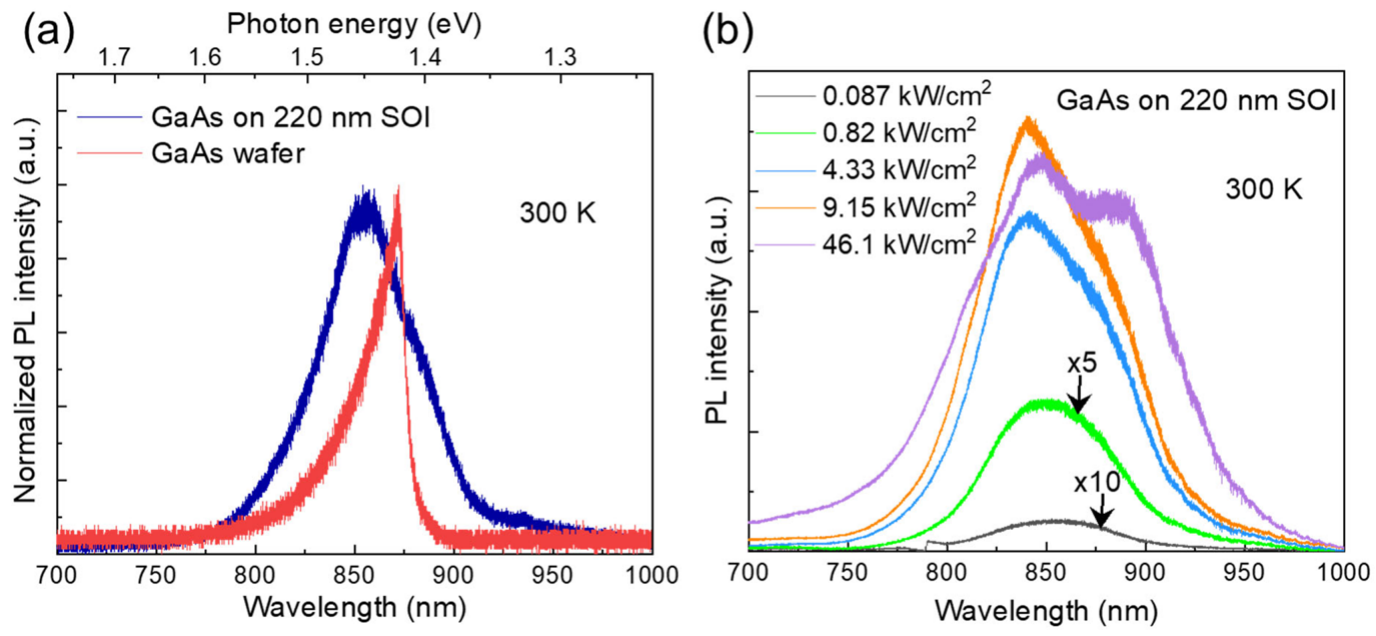
(a) Normalized PL spectra of the uncoalesced GaAs on SOI, as shown in Figure 2b, and commercial semi-insulating GaAs wafer. (b) Room temperature power dependent PL spectra of the uncoalesced GaAs on 220 nm SOI.

We also performed optical characterization of the epitaxial GaAs using room temperature microphotoluminescence (PL). A continuous-wave 660 nm laser with a ∼10 μm diameter spot was used as the excitation source. The PL emission from the coalesced GaAs (as shown in the SEM photo in Figure 2c) appears to be very dim, likely attributed to regions with grain boundaries or twinning misorientations. Instead, PL spectra of the uncoalesced GaAs (as shown by the SEM photo in Figure 2b) were measured. Figure 7a shows the comparison of normalized PL spectra from the GaAs on SOI and a reference semi-insulating (SI) undoped GaAs substrate at a low pumping power. The unintentionally doped (UID) GaAs grown via MOCVD typically exhibits n-type background doping, which could induce a blue shift in the PL peak due to the change on Fermi energy level.^35,36^ For a comprehensive PL comparison between GaAs grown on SOI and SI GaAs wafers, please refer to Figure S2. With the PL of the GaAs on SOI exhibiting a longer tail on the low-energy side, the extracted full-width-half-maximum (FWHM) (60 nm) is larger than the value (22 nm) measured from the reference GaAs wafer. We attribute the broader PL to the presence of planar defects and associated type II band transitions near the GaAs/Si interface.^37,38^ As shown by the power dependent PL of GaAs on SOI in Figure 7b, as the pumping density increases and the peak wavelength blue-shifts, the tail of the PL peak on the long-wavelength side gets more pronounced. Upon reaching a high pumping power of 46.1 kW/cm^2^, we observed a saturation in the PL peak intensity. Simultaneously, a side-peak at a more extended wavelength of 890 nm started to emerge. We associate this side-peak at the longer wavelength, particularly noticeable under high pump power, with the presence of planar defects near the Si interface.^38^

## Conclusion

To summarize, we have demonstrated a front-end patterning and epitaxy approach on Si photonics 220 nm SOI substrates, which enables direct growth of GaAs in plane to the Si device layer. By leveraging confinement of dislocations along the lateral crystalline orientation, threading dislocations generated from the Si interface can be effectively trapped within ∼250 nm distance from the Si. GaAs regions that are free of any crystalline defects can be obtained right on top of the buried oxide layer with close placement to the Si layer. Zincblende phase was confirmed from epitaxial GaAs by cross-sectional TEM investigations. The demonstrated GaAs-on-insulator platform could pave the way to realize intimately integrated III–V/Si photonic devices on the mainstream 220 nm SOI platform.

## Data Availability

The data that support the findings of this study are available on the Cardiff University Research Portal at http://doi.org/10.17035/d.2023.0287470374.

## Supporting Information Available

Additional information for lateral growth of GaAs on SOI; microphotoluminescence comparison between GaAs on SOI and native SI GaAs substrates (PDF)
