# Supplementary material for: Lateral Tunnel Epitaxy of GaAs in Lithographically Defined Cavities on 220 nm Silicon-on-Insulator
Source: Cryst Growth Des. 2023 Oct 12;23(11):7821–8. doi: 10.1021/acs.cgd.3c00633 (PMC10626574; doi:10.1021/acs.cgd.3c00633)
Supplement: Supplementary file 1 — cg3c00633_si_001.pdf [file cg3c00633_si_001.pdf]

Supplementary information

Lateral tunnel epitaxy of GaAs in lithographically defined cavities on 220 nm silicon-on-insulator

Zhao Yan [a) 1], Bogdan-Petrin Ratiu [a) 1], Weiwei Zhang [2], Oumaima Abouzaid [1], Martin Ebert [2], Graham T. Reed [2], David J. Thomson [2], and Qiang Li [b) 1]

[1] School of Physics and Astronomy, Cardiff University, Cardiff CF24 3AA, UK

[2] Optoelectronics Research Centre, University of Southampton, Southampton SO17 1BJ, UK

[a)] Zhao Yan and Bogdan-Petrin Ratiu contributed equally to this work

[b)] Tel: +44 (0)29 2087 4665, Email: LiQ44@cardiff.ac.uk

I. Lateral growth of GaAs on SOI

II. PL comparison of UID GaAs on SOI and SI GaAs wafer

III. References

I. Lateral growth of GaAs on SOI

The microscope picture of Supplementary Figure S1 displays a large view of the patterned area: The region for GaAs growth on the sample surface is confined to a small area (as indicated by the patterned area). The majority of the surface area of the sample remains either unpatterned, comprising a 220 nm Si device layer coated with SiN, or features test structures pertaining to pattern fabrication. GaAs growth is localized to the limited region in the middle the sample. For lateral growth of GaAs, the lateral width of GaAs is limited by the capability of precursor diffusions from the SiN opening into the Si seed facet for growth. Insufficient precursor diffusion can lead to inadequate growth selectivity, resulting in the formation of high-density parasitic GaAs depositions on top of the upper SiN growth mask. The thickness of GaAs is identical to the thickness of the Si device layer of the SOI substrates. In this study, we employed SOI substrates with a Si device layer thickness of 220 nm. However, SOI substrates with different Si device layer thicknesses can also be utilized. A thicker Si device layer can create a thicker tunnel (a larger distance between the upper SiN layer and the lower buried oxide layer), which may facilitate precursor diffusion within the tunnel.

Prior to growth, high temperature annealing at 800 °C for 20 minutes was performed. The 800 °C annealing temperature is typically utilized in conventional III-V thin film growth on planar Si substrates for oxide desorption. Prior work involving selective area growth of III-V on Si suggests that decreasing the annealing duration to 15 minutes or 10 minutes could also be feasible. [1, 2] The GaAs was grown at a single temperature at 680 °C. We have also attempted the two-step growth method including a low temperature (LT) GaAs nucleation layer and a high temperature (HT) GaAs main layer, which, however, led to high-density parasitic GaAs deposition on the SiN dielectric mask. We found that this inadequate growth selectivity primarily stems from the LT GaAs layer, in which the TEGa adatoms exhibit limited diffusion at the nucleation

temperature. Without the GaAs nucleation layer, growth selectivity can be substantially improved. The microscope picture of Figure S1 below presents the sample surface after the GaAs growth without the LT GaAs.

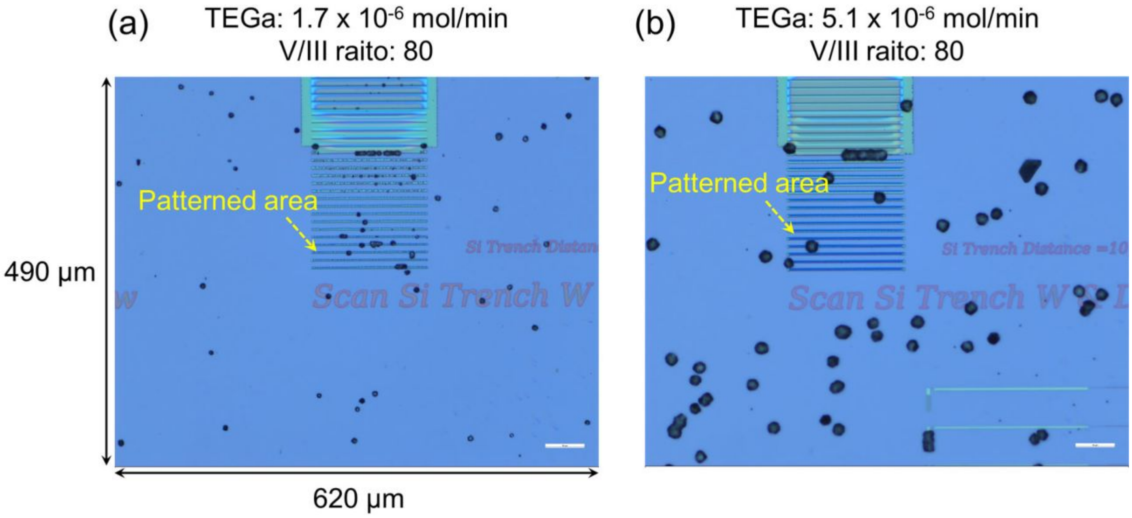

Figure S1. Large-view optical microscope images after the GaAs growth without the low temperature GaAs nucleation layer.

As shown by the SEM photos of Figure 2 in the manuscript, the GaAs shows a 3D initial growth without the low temperature nucleation layer, and both straight and bumpy end facets were formed at the growth front of GaAs (Figure 2c). In order to achieve defect-free coalescence and extend the growth for very long templates, we consider two directions to achieve a uniform GaAs growth front. It has been shown from SEM images that GaAs islands can be formed with two vertical {110} facets on both sides, as marked by the white dotted line in Figure 2b. As previously demonstrated, coalescence between the union of {110} side-facets can result in defect-free coalesced regions. [2-4] This is likely due to the non-polar attribute of the {110} facets of zincblende crystal and the well-aligned crystalline orientations between two vertical {110} facets. Therefore, growth optimization can be performed to form uniform {110} side-facets and uniform

growth fronts, which can be utilized to coalesce the GaAs into continuous slabs. The other option may involve the use of nucleation layers made of InP or InAs, both of which can provide better growth selectivity, dense nucleation islands and longer diffusion length compared to GaAs at low growth temperatures. [5]

II. PL comparison of UID GaAs on SOI and SI GaAs wafer

Given the different material volume between the GaAs on 220 nm SOI and the GaAs wafer, we employed an extremely low pumping power to probe the unintentionally-doped (UID) GaAs on a 220 nm SOI as well as the reference semi-insulating (SI) GaAs substrate, as illustrated in Figure S2a. Comparative analyses at increasing pump powers are presented in Figures S2 (b-e). Interestingly, under low pump power, the GaAs on SOI manifests a substantially elevated PL intensity in comparison to the GaAs wafer (Figure S2a). As the pump power increases, the difference of the PL intensity between the GaAs on SOI and GaAs wafer decreases (see Figure S2b-e below). We attribute this phenomenon to two potential reasons: First, the n-type background doping in UID GaAs grown by MOCVD can contribute to enhanced PL intensity. [6, 7] Second, the distinct structural differences between the GaAs on SOI and the GaAs wafer can result in different pumping conditions. In the case of GaAs on SOI, it is feasible that multiple round-trip reflections of the pump light within the GaAs may occur. Such reflections are characterized by the pump light oscillating within the GaAs structure, facilitated by strong reflection at both the top GaAs/SiN and bottom GaAs/SiO_2 interfaces. This could be advantageous for the PL intensity of GaAs on SOI, especially when the pump power is low.

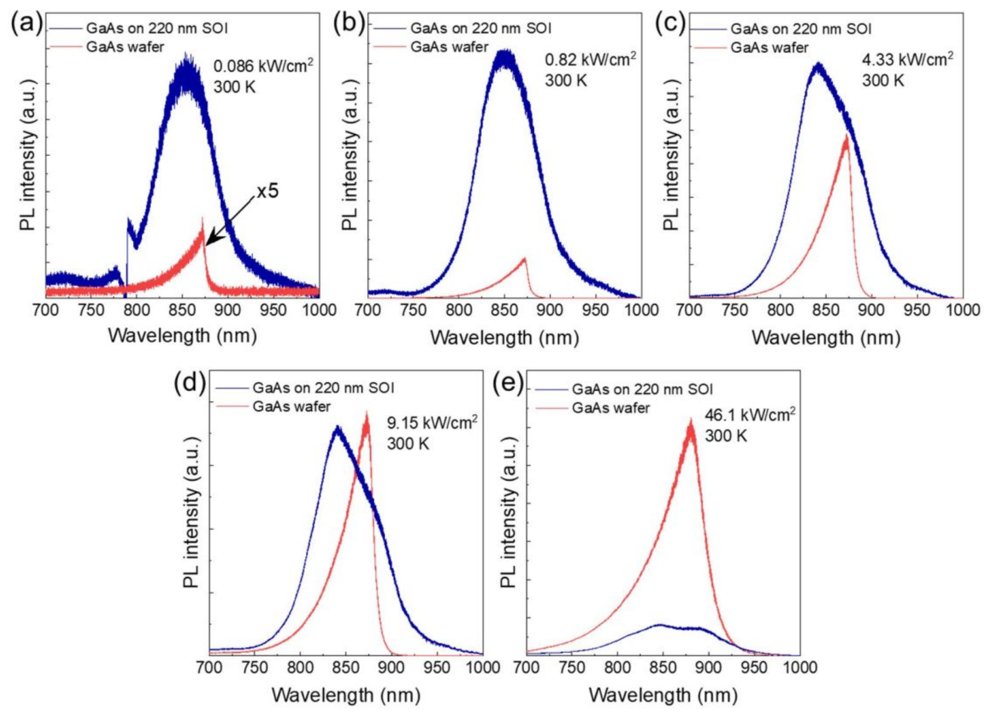

Figure S2. (a)-(e) Power-dependent PL comparison between the uncoalesced GaAs on SOI and the semi-insulating GaAs wafer. A PL stitch of the GaAs on SOI curve is observed in Figure (a) at low PL intensity.

III. References:

[1] Z. Yan, Y. Han, and K. M. Lau, "Multi-heterojunction InAs/GaSb nano-ridges directly grown on (001) Si", Nanotechnology 31 (2020) 345707 (7pp).

[2] Z. Yan, Y. Han, and K. M. Lau, "InAs nano-ridges and thin films grown on (001) silicon substrates", J. Appl. Phys. 128, 035302 (2020).

[3] Q. Li, B. Lai, and K. M. Lau, "Epitaxial growth of GaSb on V-grooved Si (001) substrates with an ultrathin GaAs stress relaxing layer", Appl. Phys. Lett. 111, 172103 (2017).

[4] P. Staudinger, K. E. Moselund, and H. Schmid, "Exploring the Size Limitations of Wurtzite III−V Film Growth", Nano Lett. 2020, 20, 686−693.

[5] Z. Yan, Y. Han, L. Lin, Y. Xue, C. Ma, ... and K. M. Lau, "A monolithic InP/SOI platform for integrated photonics." Light Sci. Appl. 10, 200 (2021).

[6] C. Lee, N. Lee, K. Lee, J. Kim, ... and H. Lim, "Room-temperature photoreflectance and photoluminescence of heavily Si-doped GaAs", J. Appl. Phys 77 (12), 15 June 1995.

[7] Y. Fu, M. Willander, G. Chen, Y. Ji, and W. Lu, "Photoluminescence spectra of doped GaAs films", Appl. Phys. A 79, 619–623 (2004).
